# Effects of inappropriate cause-of-death certification on mortality from cardiovascular disease and diabetes mellitus in Tonga

**DOI:** 10.1186/s12889-023-17294-z

**Published:** 2023-12-01

**Authors:** Carah A. Figueroa, Christine L. Linhart, Catherine Dearie, Latu E. Fusimalohi, Sioape Kupu, Stephen L. Morrell, Richard J. Taylor

**Affiliations:** 1 Statistics for Development Division, Pacific Community, Nouméa, New Caledonia; 2 School of Population Health, University of New South Wales, UNSW, Sydney, Australia; 3 Ministry of Health, Nuku’alofa, Tonga

**Keywords:** Cardiovascular disease, Diabetes, Adult mortality, Cause of death, Death certification, ICD coding, Tonga

## Abstract

**Background:**

Cardiovascular disease (CVD) and diabetes mellitus are major health issues in Tonga and other Pacific countries, although mortality levels and trends are unclear. We assess the impacts of cause-of-death certification on coding of CVD and diabetes as underlying causes of death (UCoD).

**Methods:**

Tongan records containing cause-of-death data (2001–2018), including medical certificates of cause-of-death (MCCD), had UCoD assigned according to International Classification of Diseases 10th revision (ICD-10) coding rules. Deaths without recorded cause were included to ascertain total mortality. Diabetes and hypertension causes were reallocated from Part 1 of the MCCD (direct cause) to Part 2 (contributory cause) if potentially fatal complications were not recorded, and an alternative UCoD was assigned. Proportional mortality by cause based on the alternative UCoD were applied to total deaths then mortality rates calculated by age and sex using census/intercensal population estimates. CVD and diabetes mortality rates for unaltered and alternative UCoD were compared using Poisson regression.

**Results:**

Over 2001–18, in ages 35–59 years, alternative CVD mortality was higher than unaltered CVD mortality in men (*p* = 0.043) and women (*p* = 0.15); for 2010–18, alternative versus unaltered measures in men were 3.3/10^3^ (95%CI: 3.0–3.7/10^3^) versus 2.9/10^3^ (95%CI: 2.6–3.2/10^3^), and in women were 1.1/10^3^ (95%CI: 0.9–1.3/10^3^) versus 0.9/10^3^ (95%CI: 0.8–1.1/10^3^). Conversely, alternative diabetes mortality rates were significantly lower than the unaltered rates over 2001–18 in men (*p* < 0.0001) and women (*p* = 0.013); for 2010–18, these measures in men were 1.3/10^3^ (95%CI: 1.1–1.5/10^3^) versus 1.9/10^3^ (95%CI: 1.6–2.2/10^3^), and in women were 1.4/10^3^ (95%CI: 1.2–1.7/10^3^) versus 1.7/10^3^ (95%CI: 1.5–2.0/10^3^). Diabetes mortality rates increased significantly over 2001–18 in men (unaltered: *p* < 0.0001; alternative: *p* = 0.0007) and increased overall in women (unaltered: *p* = 0.0015; alternative: *p* = 0.014).

**Conclusions:**

Diabetes reporting in Part 1 of the MCCD, without potentially fatal diabetes complications, has led to over-estimation of diabetes, and under-estimation of CVD, as UCoD in Tonga. This indicates the importance of controlling various modifiable risks for atherosclerotic CVD (including stroke) including hypertension, tobacco use, and saturated fat intake, besides obesity and diabetes. Accurate certification of diabetes as a direct cause of death (Part 1) or contributory factor (Part 2) is needed to ensure that valid UCoD are assigned. Examination of multiple cause-of-death data can improve understanding of the underlying causes of premature mortality to better inform health planning.

**Supplementary Information:**

The online version contains supplementary material available at 10.1186/s12889-023-17294-z.

## Introduction

Tonga is a South Pacific Island nation with a 2016 census population of 100,651, of which 39% was aged ≤ 15 years, and 9% ≥ 60 years [1]. Non-communicable diseases (NCDs), in particular cardiovascular disease (CVD), diabetes mellitus (diabetes), and cancers, have been widely reported as the major causes of morbidity and premature adult mortality in Tonga [2–10], despite gaps in data on NCD mortality, incidence, prevalence, and causal risk 

Diabetes and hypertension are major risk factors for atherosclerotic CVD occurrence and mortality. If poorly controlled, both conditions can also directly lead to death from specific complications such as diabetic coma or diabetic renal disease with chronic renal failure, and hypertensive heart diseases with heart failure or hypertensive renal disease with renal failure. In cases of deaths from specific diabetic complications, the medical certifier would appropriately place diabetes in Part 1 of the medical certificate of cause of death (MCCD) (see Additional File 1: Fig. S1A). The coder would then select diabetes as the underlying cause of death (UCoD), in the absence of cancer or an external injury, according to the International Statistical Classification of Diseases and Related Health Problems 10^th^ revision (ICD-10) coding rules [11, 12]. Likewise, for deaths from hypertensive complications, hypertension would be appropriately placed in Part 1. For atherosclerotic CVD deaths without potentially fatal diabetic or hypertensive complications, diabetes and hypertension should be reported in Part 2 of the MCCD, as contributory causes. When cancers or external causes are reported in Part 1 of the MCCD, they are to be accepted as the UCoD with a few exceptions [12].

Inappropriate placement of uncomplicated diabetes, or diabetes with non-fatal complications, in Part 1 of the MCCD, and consequent assignment of diabetes as the UCoD, can yield spurious levels of diabetes mortality in reported mortality statistics. This can lead to under-estimation of the contribution of atherosclerotic CVD, as has been demonstrated in countries such as Fiji and Mauritius [13], Australia and the United States [14], and Taiwan [15]. Given the negligible impact of misplacement of uncomplicated diabetes on the estimation of non-CVD major causes of death, particularly cancer or injury deaths, this study focuses on changes in the levels of mortality due to CVD and diabetes. A detailed consideration of other main causes of death would significantly extend the length of the article and would deflect from the main analysis and findings.

The objective of this study is to investigate the consequences of certification practices and subsequent ICD-10 coding on CVD and diabetes mortality levels and trends in Tonga. We hypothesise that levels of diabetes as UCoD may be over-estimated and consequently trends in CVD as UCoD under-estimated, partly as a consequence of misclassification of the UCoD to diabetes, rather than real changes in these causes of death.

## Methods

### Data collection

Unit records containing direct and contributing causes-of-death were obtained, including: (i) MCCDs held at the Tonga Ministry of Health (MoH), which are issued for hospital deaths and notified community deaths; (ii) community nursing reports through the Reproductive Health Services System; and (iii) hospital separations as death from Tonga’s main hospital (Vaiola). Civil Registry records managed by the Ministry of Justice [16, 17] do not report cause-of-death, but were employed to enumerate total (all-causes) mortality [16]. At the time of this study, mortality data were available from these four sources for 2010–2018, with MCCDs also available for earlier years, 2001–2009. Methods for record linkage and assessment of reporting completeness are detailed elsewhere [16]. MCCDs, community nursing reports and hospital discharge records collectively captured 97% of reported deaths in 2010–2018 [16].

### Assignment of underlying cause of death

Mortality coders in Tonga manually code MCCDs (see Additional File 1: Fig. S1B) using ICD-10 version 4. Automated coding system Iris [18] was used for standardised selection of the UCoD, according to ICD-10 rules [11, 12]. Iris is used internationally, including in the Asia–Pacific region (Australia, New Zealand, Fiji). Electronic MCCDs consistent with the international MCCD were prepared from the Tongan MCCDs, community nursing reports and hospital discharge death records, then processed in Iris v5.7.0 [18]. Over 80% of the Tongan MCCDs in 2010–18 provided conditions only for Part 1 of the Iris MCCDs. Hence, all conditions recorded in community nursing reports and hospital discharge records were placed in Part 1 of the Iris MCCDs. Ambiguous causal sequences, injury deaths and maternal deaths that Iris rejected were manually reviewed (3% in ages 35–74 years, 2% in all ages).

### Identification and adjustment of possible inappropriate causal sequences

For MCCDs containing diabetes (type 1, type 2, unspecified) or hypertension in Part 1, codes were either: (i) retained in Part 1 as a direct cause-of-death if specific potentially fatal complications were present; or (ii) relocated to Part 2 as a contributory cause if the specific complications were absent. Table S1 presents the criteria and rationale for the code placement (see Additional file 2). After re-assignment, the causal sequences and UCoD are described herein as ‘alternative’. Figs. S2 and S3, illustrate examples (see Additional files 3 and 4). Tables S2 and S3 present the ICD-10 coding rules for selecting diabetes and hypertension as the UCoD when they are reported in Part 1 with other conditions (see Additional files 5 and 6).

### Statistical analysis

The UCoD from both unaltered and alternative cause-of-death sequences were aggregated to disease categories: endocrine, nutritional and metabolic diseases (ICD-10 codes E00-E90), diabetes (codes E10-E14), circulatory diseases (codes I00-I99), CVD (codes I10-I69), hypertensive diseases (codes I10-I13), ischaemic heart diseases (IHD) (codes I20-I25), other heart diseases (codes I26-I51), cerebrovascular diseases (codes I60-I69), and all other causes. Deaths coded to ill-defined causes (codes R00-R99) were proportionally redistributed across known causes. Deaths coded to ill-defined CVD (such as heart failure, sudden cardiac arrest, atherosclerosis) were proportionally redistributed to age-dependent CVD and chronic respiratory disease categories, following published redistribution methods [19–22].

Cause-specific proportional mortality and mortality rates were calculated by sex and age group (35–59 and 60–74 years), and aggregated to six triennia (2001–03, 2004–06, 2007–09, 2010–12, 2013–15, and 2016–18) (‘triennial proportional mortality/ rates’) and two 9-year periods (2001–09 and 2010–18) (‘period proportional mortality/ rates’) to reduce stochastic and other variation. Unaltered and alternative measures were compared. Exact binomial (Clopper-Pearson) confidence intervals (95%CI) for proportional mortality were calculated. Triennial proportional mortality by sex were assessed by Mantel–Haenszel χ^2^ test for trend.

Cause-specific mortality rates were calculated by applying proportional mortality to total deaths. See Additional File 7 for calculations of the rate numerators. The rate denominator populations were derived from linear interpolation or projections of age- and sex-specific populations from the 2006, 2011 and 2016 censuses [1, 23, 24]. For assessment of time trends, mortality rates were directly age-standardised to the Tonga 2011 census population. Poisson 95%CI for the rates [25] and Dobson Poisson CI for age-standardised rates [26] were calculated. Differences in period mortality rates and period trends in triennial rates were assessed by Poisson regression of age-specific counts of deaths (offset by the log of the denominator population) using the GENMOD procedure in SAS 9.4 software (SAS Institute Inc.). All statistical analyses were conducted in SAS 9.4 (SAS Institute Inc.) and Excel (2016) (Microsoft Corporation).

## Results

### Reported deaths and total mortality

The 2001–18 MCCD dataset included 5,679 deaths in ages 35–74 years (54%). The 2010–18 cause-of-death dataset (MCCD, hospital discharge, and community nursing reports) included 3,984 deaths aged 35–74 years (55%), of which 23% had no MCCD available, but were reported in the other sources. Deaths in ages 35–74 years comprised 59% men, 41% women. In ages 35–59 years, age-standardised all-cause mortality increased in men (*p* < 0.0001) from 8.8 deaths/10^3^ (95%CI: 8.2–9.4/10^3^) in 2001–09 to 10.1/10^3^ in 2010–18 (95%CI: 9.5-10.7/10^3^) (Table 1), while in women, mortality was around 7.0/10^3^ across both periods (Table 2). All-cause mortality in ages 60–74 years remained stable over the period.

**Table 1:** Cardiovascular diseases and diabetes proportional mortality (%) and mortality rates (per 1,000) in men aged 35–59 years and 60–74 years, Tonga, 2001–2009 and 2010–2018: unaltered versus alternative underlying cause of death certification^a^

Cause^b^	Number (proportional mortality, %)				Mortality rate (/1,000) (95%CI)			
**Period:**	**2001–09**		**2010–18**		**2001–09**		**2010–18**	
**Certification**^**a**^**:**	**Unalt**^**a**^	**Alt**^**a**^	**Unalt**^**a**^	**Alt**^**a**^	**Unalt**^**a**^	**Alt**^**a**^	**Unalt**^**a**^	**Alt**^**a**^
**35–59 years**								
**Circulatory**^**c**^	**210 (36)**	**225 (38)**	**290 (30)**	**331 (34)**	**3.1** **(2.8–3.5)**	**3.3** **(3.0–3.7)**	**3.0** **(2.7–3.3)**	**3.4** **(3.1–3.8)**
IHD	157 (27)	169 (29)	203 (21)‡	234 (24)	2.3 (2.0–2.7)	2.5 (2.2–2.8)	2.1 (1.8–2.4)	2.4 (2.1–2.7)
Other heart	16 (2.7)	16 (2.7)	24 (2.5)	29 (3.0)	0.2 (0.1–0.4)	0.2 (0.1–0.4)	0.3 (0.2–0.4)	0.3 (0.2–0.4)
Hypertensive	22 (3.7)	25 (4.2)	25 (2.5)	27 (2.7)	0.3 (0.2–0.5)	0.4 (0.3–0.5)	0.3 (0.2–0.4)	0.3 (0.2–0.4)
Cerebrovascular	10 (1.7)	10 (1.7)	28 (2.9)	31 (3.2)	0.2 (0.08–0.3)	0.2 (0.08–0.3)	0.3 (0.2–0.4)‡	0.3 (0.2–0.4)‡
**Endocrine**	**95 (16)**	**78 (13)**	**206 (21)‡**	**146 (15)**	**1.4** **(1.2–1.7)**	**1.2** **(0.9–1.4)**	**2.1** **(1.8–2.4)‡**	**1.5** **(1.3–1.8)‡**
Diabetes	**89 (15)**	**71 (12)**	**185 (19)‡**	**123 (13)**	**1.3** **(1.1–1.6)**	**1.1** **(0.9–1.3)**	**1.9** **(1.6–2.2)‡**	**1.3** **(1.1–1.5)**
***Other causes***	287 (48)	289 (49)	479 (49)	498 (51)	4.2 (3.8–4.7)	4.3 (3.9–4.7)	5.0 (4.6–5.4)	5.2 (4.8–5.6)
**All causes**^**d**^	**592 (100)**		**975 (100)**		**8.8 (8.2–9.4)**		**10.1 (9.5–10.7)‡**	
Ill-def, unk^d^	50 (7.8)		85 (8.0)		—		—	
Cardiac arrest^c^	56 (8.7)		35 (3.3)		—		—	
Other ill-def CVD^c^	17 (2.6)	17 (2.6)	14 (1.3)	17 (1.6)	—		—	
**60–74 years**								
**Circulatory**^**c**^	**282 (32)**	**315 (35)**	**328 (28)**	**376 (32)**	**14.5** **(13.1–16.0)**	**16.1** **(14.6–17.7)**	**13.3** **(12.0–14.7)**	**15.3** **(13.8–16.8)**
IHD	**160 (18)**	**177 (20)**	**194 (17)**	**227 (20)**	**8.2** **(7.2–9.4)**	**9.1** **(8.0–10.2)**	**7.8** **(6.8–8.9)**	**9.2** **(8.1–10.4)**
Other heart	39 (4.3)	43 (4.9)	19 (1.7)‡	24 (2.0)‡	2.0 (1.5–2.6)	2.2 (1.7–2.8)	0.8 (0.5–1.2)‡	1.0 (0.6–1.4)‡
Hypertensive	40 (4.5)	42 (4.7)	47 (4.0)	37 (3.2)	2.0 (1.5–2.6)	2.1 (1.6–2.7)	1.9 (1.4–2.5)	1.5 (1.1–2.1)
Cerebrovascular	**39 (4.4)**	**48 (5.4)**	**63 (5.4)**	**83 (7.1)**	**2.0** **(1.5–2.6)**	**2.5** **(1.9–3.1)**	**2.6** **(2.0–3.3)**	**3.4** **(2.7–4.2)**
**Endocrine**	**146 (16)**	**104 (12)**	**252 (22)‡**	**181 (16)‡**	**7.4** **(6.4–8.5)**	**5.3** **(4.5–6.2)**	**10.2** **(9.0–11.5)‡**	**7.3** **(6.3–8.4)‡**
Diabetes	**135 (15)**	**92 (10)**	**238 (20)‡**	**165 (14)‡**	**6.9** **(5.9–7.9)**	**4.7** **(3.9–5.6)**	**9.7** **(8.5–10.9)‡**	**6.7** **(5.7–7.7)‡**
**Other causes**	460 (52)	469 (53)	583 (50)	606 (52)	23.6 (21.9–25.5)	24.2 (22.3–26.0)	23.8 (22.0–25.7)	24.8 (22.9–26.7)
**All causes**^**d**^	**888 (100)**		**1163 (100)**		**45.6 (43.1–48.1)**		**47.3 (44.8–49.9)**	
Ill-def, unk^d^	63 (6.6)		113 (11.4)		—		—	
Cardiac arrest^c^	70 (7.4)		43 (4.4)		—		—	
Other ill-def CVD^c^	46 (4.8)	48 (5.0)	26 (2.0)	28 (2.2)	—		—	

Proportional mortality (PM) are based on the underlying cause of death (UCoD) from medical certificates of cause of death (MCCD). UCoD from CoD in community nursing or hospital discharge records in 2010–18 were included in the estimation of PM for 2010–18. Differences in PM assessed by chi-square (χ2) tests, Dash (—), not applicable. Mortality rates are directly age-standardised to the Tonga 2011 census population. Difference in rates assessed by Poisson regression; ‡ Difference between 2001–09 and 2010–18 (*p* < 0.05); Dash (—), not applicable. a Unalt, unaltered UCoD certification; Alt, alternative plausible UCoD certification following criteria for placement of diabetes and hypertension codes in Part 1 or Part 2. The 2001–09 mortality rate numerators are the 2001–09 deaths adjusted by the reporting completeness of MCCD in 2010–18. The 2010–18 rate numerators are estimated by applying the 2010–18 PM to the total deaths in 2010–18 ascertained previously [16]. Difference between unaltered and alternative UCoD (*p* < 0.05) are shown in bold. b International Classification of Diseases, 10th revision (ICD-10) chapters Circulatory diseases (Circulatory) (ICD-10 codes I00–I99) and Endocrine, nutritional, and metabolic diseases (Endocrine) (E00–E90) are shown in bold. Circulatory includes cardiovascular diseases (CVD): IHD: ischaemic heart disease (I20–I25); Other heart: other heart diseases (I26–I51); Hypertensive diseases (I10–I15); Cerebrovascular diseases (I60–I69). Endocrine includes Diabetes: diabetes mellitus (E10–E14). Other causes includes remaining chapters. Ill-def, unk: ill-defined and unknown causes; Other ill-def CVD: other ill-defined cardiovascular disease. c Circulatory excludes ill-defined cardiovascular conditions (I46.-, I47.2, I49.0, I50, I51.4, I51.5, I51.6, I51.9, I70.9). These codes were redistributed proportionately as follows: in ages 35–49 years: to IHD and ‘other heart diseases’ (cardiomyopathy, myocarditis, endocarditis); in ages ≥ 50 years: to IHD, cardiomyopathy, hypertensive diseases, chronic respiratory disease. d Deaths coded to ill-defined conditions (R00–R94) and unknown causes (R96–R99) were redistributed proportionately to other causes within age-sex groups

**Table 2:** Cardiovascular diseases and diabetes proportional mortality (%) and mortality rates (per 1,000) in women aged 35–59 years and 60–74 years, Tonga, 2001–2009 and 2010–2018: unaltered versus alternative underlying cause of death certification^a^

Cause^b^	Number (proportional mortality, %)				Mortality rate (/1,000) (95%CI)			
**Period:**	**2001–09**		**2010–18**		**2001–09**		**2010–18**	
**Certification**^**a**^**:**	**Unalt**^**a**^	**Alt**^**a**^	**Unalt**^**a**^	**Alt**^**a**^	**Unalt**^**a**^	**Alt**^**a**^	**Unalt**^**a**^	**Alt**^**a**^
**35–59 years**								
**Endocrine**	**102 (22)**	**80 (17)**	**178 (26)‡**	**155 (22)‡**	**1.5** **(1.2–1.7)**	**1.2** **(1.0–1.4)**	**1.8** **(1.6–2.0)**	**1.6** **(1.3–1.8)‡**
Diabetes	**95 (20)**	**73 (15)**	**168 (24)‡**	**143 (20)‡**	**1.4** **(1.2–1.6)**	**1.1** **(0.9–1.3)**	**1.7** **(1.5–2.0)**	**1.4** **(1.2–1.7)‡**
**Circulatory**^**c**^	97 (21)	115 (24)	106 (15)‡	119 (17)‡	1.5 (1.2–1.7)	1.7 (1.4–2.0)	1.1 (0.9–1.3)‡	1.2 (1.0–1.4)‡
IHD	30 (6.3)	40 (8.4)	39 (5.6)	45 (6.5)	0.4 (0.3–0.6)	0.6 (0.4–0.7)	0.4 (0.3–0.5)	0.5 (0.3–0.6)
Other heart	18 (3.8)	20 (4.3)	15 (2.1)	17 (2.4)	0.3 (0.2–0.4)	0.3 (0.2–0.4)	0.2 (0.1–0.2)	0.2 (0.1–0.3)
Hypertensive	25 (5.4)	27 (5.7)	17 (2.4)‡	17 (2.4)‡	0.4 (0.3–0.5)	0.4 (0.3–0.6)	0.2 (0.1–0.3)‡	0.2 (0.1–0.3)‡
Cerebrovascular	16 (3.4)	19 (4.0)	23 (3.3)	26 (3.7)	0.2 (0.1–0.3)	0.3 (0.2–0.4)	0.2 (0.2–0.3)	0.3 (0.2–0.4)
**Other causes**	273 (58)	277 (59)	414 (59)	424 (61)	4.0 (3.6–4.4)	4.0 (3.7–4.5)	4.2 (3.8–4.6)	4.3 (3.9–4.7)
**All causes**^**d**^	**472 (100)**		**698 (100)**		**6.9 (6.4–7.4)**		**7.0 (6.6–7.6)**	
Ill-def, unk^d^	46 (8.9)		60 (7.9)		—		—	
Cardiac arrest^c^	25 (4.8)		4 (0.58)		—		—	
Other ill-def CVD^c^	15 (2.9)	16 (3.1)	5 (0.66)	6 (0.79)	—		—	
**60–74 years**								
**Endocrine**	**190 (33)**	**148 (25)**	**277 (34)**	**222 (27)**	**10.1** **(9.0–11.3)**	**7.8** **(6.8–8.9)**	**10.1** **(9.0–11.3)**	**8.0** **(7.1–9.2)**
Diabetes	**187 (32)**	**143 (25)**	**264 (32)**	**205 (25)**	**9.9** **(8.8–11.2)**	**7.6** **(6.6–8.7)**	**9.6** **(8.5–10.8)**	**7.5** **(6.5–8.5)**
**Circulatory**^**c**^	**137 (23)**	**174 (30)**	**167 (21)**	**206 (25)**	**7.4** **(6.4–8.4)**	**9.4** **(8.3–10.5)**	**6.3** **(5.4–7.2)**	**7.6** **(6.6–8.6)‡**
IHD	**47 (8.1)**	**63 (11)**	**65 (8.0)**	**82 (10)**	**2.5** **(2.0–3.2)**	**3.4** **(2.8–4.2)**	**2.4** **(1.8–3.0)**	**3.0** **(2.4–3.7)**
Other heart	16 (2.7)	20 (3.4)	10 (1.3)	19 (2.3)	0.9 (0.6–1.3)	1.1 (0.7–15)	0.5 (0.3–0.8)	0.7 (0.4–1.0)
Hypertensive	34 (5.8)	41 (7.1)	38 (4.7)	37 (4.6)	1.8 (1.3–2.4)	2.2 (1.7–2.8)	1.4 (1.0–1.9)	1.4 (1.0–1.8)‡
Cerebrovascular	34 (5.9)	43 (7.4)	46 (5.7)	59 (7.2)	1.8 (1.4–2.4)	2.3 (1.8–2.9)	1.7 (1.3–2.2)	2.2 (1.7–2.7)
**Other causes**	254 (44)	259 (45)	370 (45)	386 (47)	13.6 (12.3–15.0)	13.9 (12.5–15.3)	13.3 (12.1–14.7)	14.0 (12.8–15.5)
**All causes**^**d**^	**581 (100)**		**814 (100)**		**31.1 (29.1–33.2)**		**29.7 (27.8–31.7)**	
Ill-def, unk^d^	66 (10.2)		76 (8.5)		—		—	
Cardiac arrest^c^	24 (3.7)		8 (0.90)		—		—	
Other ill-def CVD^c^	28 (4.3)	28 (4.3)	15 (1.7)	15 (1.7)	—		—	

Proportional mortality (PM) are based on the underlying cause of death (UCoD) from medical certificates of cause of death (MCCD). UCoD from CoD in community nursing or hospital discharge records in 2010–18 were included in the estimation of PM for 2010–18. Differences in PM assessed by chi-square (χ2) tests, Dash (—), not applicable. Mortality rates are directly age-standardised to the Tonga 2011 census population. Difference in rates assessed by Poisson regression; ‡ Difference between 2001–09 and 2010–18 (*p* < 0.05); Dash (—), not applicable. a Unalt, unaltered UCoD certification; Alt, alternative plausible UCoD certification following criteria for placement of diabetes and hypertension codes in Part 1 or Part 2. The 2001–09 mortality rate numerators are the 2001–09 deaths adjusted by the reporting completeness of MCCD in 2010–18. The 2010–18 rate numerators are estimated by applying the 2010–18 PM to the total deaths in 2010–18 ascertained previously [16]. Difference between unaltered and alternative UCoD (*p* < 0.05) are shown in bold. b International Classification of Diseases, 10th revision (ICD-10) chapters Circulatory diseases (Circulatory) (ICD-10 codes I00–I99) and Endocrine, nutritional, and metabolic diseases (Endocrine) (E00–E90) are shown in bold. Circulatory includes cardiovascular diseases (CVD): IHD: ischaemic heart disease (I20–I25); Other heart: other heart diseases (I26–I51); Hypertensive diseases (I10–I15); Cerebrovascular diseases (I60–I69). Endocrine includes Diabetes: diabetes mellitus (E10–E14). Other causes includes remaining chapters. Ill-def, unk: ill-defined and unknown causes; Other ill-def CVD: other ill-defined cardiovascular disease. c Circulatory excludes ill-defined cardiovascular conditions (I46.-, I47.2, I49.0, I50, I51.4, I51.5, I51.6, I51.9, I70.9). These codes were redistributed proportionately as follows: in ages 35–49 years: to IHD and ‘other heart diseases’ (cardiomyopathy, myocarditis, endocarditis); in ages ≥ 50 years: to IHD, cardiomyopathy, hypertensive diseases, chronic respiratory disease. d Deaths coded to ill-defined conditions (R00–R94) and unknown causes (R96–R99) were redistributed proportionately to other causes within age-sex groups

The leading UCoDs in ages 35–74 years were CVD, cancers, and diabetes, however CVD and diabetes mortality estimated from the alternative UCoD certification differed from that estimated from unaltered certification.

### Cardiovascular diseases mortality

Based on unaltered cause-of-death certification, major CVDs caused 35% (95%CI: 31–38%) of deaths in 2001–09 and 29% (95%CI: 26–31%) in 2010–18 in men aged 35–59 years; 70–75% of these were IHD (Table 1). Unaltered CVD proportional mortality among these men was higher than in older men, 60–74 years, in both periods. Based on alternative CoD certification, CVD proportional mortality were 3–4% points higher than the unaltered proportional mortality in men aged 35–59 years (*p* = 0.029) and 60–74 years (*p* = 0.0054). In women, based on unaltered certification, CVDs caused 19% (95%CI: 16–22%) of deaths in 2001–09 and 13% (95%CI: 11–16%) in 2010–18 in ages 35–59 years; one third of these were IHD (Table 2). Unaltered CVD proportional mortality among these women was lower than in older women, 60–74 years, in both periods. Based on alternative cause-of-death certification, CVD proportional mortality was minimally higher than the unaltered proportional mortality in ages 35–59 years, while 5–6% points higher in ages 60–74 years (*p* = 0.0019).

**Fig. 1:**
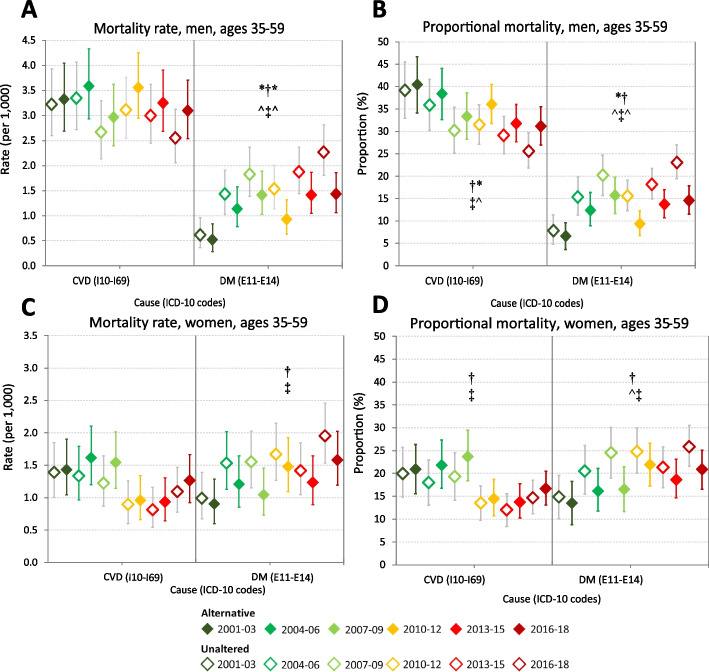
Cardiovascular diseases and diabetes proportional mortality (%) and mortality rates (per 10^3^), adults aged 35–59 years, by sex, Tonga, 2001–2018: unaltered versus alternative underlying cause of death certification^a^. Mortality rates per 1000 population by sex are directly age-standardised to the Tonga 2011 census population. Error bars reflect 95% confidence intervals. CVD (cardiovascular disease): ICD-10 codes I10–I69 (excludes cardiac arrest, heart failure and other ill-defined heart diseases which were redistributed); DM (diabetes mellitus): E10–E14^a^ Alternative: Full-coloured markers. Proportional mortality (PM) and mortality rates are based on alternative plausible certification following criteria for placement of diabetes and hypertension codes in Part 1 or Part 2 of medical certificates of cause-of-death (MCCD). The 2001–09 triennial mortality rate numerators are the 2001–09 deaths adjusted by the reporting completeness of MCCD in 2010–18. The 2010–18 triennial rate numerators are estimated by applying the 2010–18 PM to the total deaths in 2010–18 ascertained from linkage of mortality data sources [16]. Unaltered: Unfilled markers. PM and rates are based on unaltered certification PM trend assessed by Mantel–Haenszel χ2 test for trend; rates trend assessed by Poisson regression. †Trend in alternative PM or rate over triennia between 2001 and 2018 (*p* < 0.05, df = 5): *Trend in alternative PM or rate over triennia within 2001–09 or 2010–18 (*p* < 0.05, df = 2) ‡Trend in unaltered PM or rate over triennia between 2001 and 2018 (*p* < 0.05, df = 5) ^Trend in unaltered PM or rate over triennia within 2001–09 or 2010–18 (*p* < 0.05, df = 2)

**Fig. 2:**
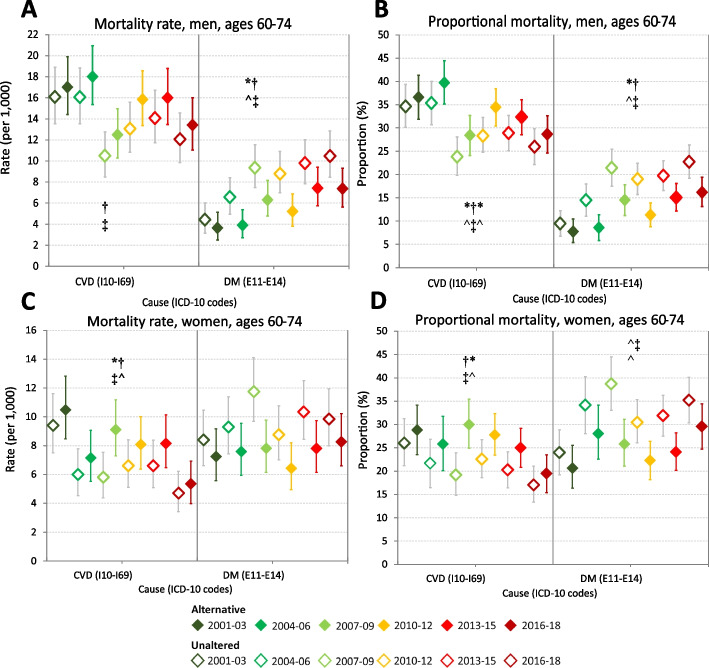
Cardiovascular diseases and diabetes proportional mortality (%) and mortality rates (per 10^3^) adults aged 60–74 years, by sex, Tonga, 2001–2018: unaltered versus alternative underlying cause of death certification^a^. Mortality rates per 1000 population by sex are directly age-standardised to the Tonga 2011 census population. Error bars reflect 95% confidence intervals. CVD (cardiovascular disease): ICD-10 codes I10–I69 (excludes cardiac arrest, heart failure and other ill-defined heart diseases which were redistributed); DM (diabetes mellitus): E10–E14^a^ Alternative: Full-coloured markers. Proportional mortality (PM) and mortality rates are based on alternative plausible certification following criteria for placement of diabetes and hypertension codes in Part 1 or Part 2 of medical certificates of cause-of-death (MCCD). The 2001–09 triennial mortality rate numerators are the 2001–09 deaths adjusted by the reporting completeness of MCCD in 2010–18. The 2010–18 triennial rate numerators are estimated by applying the 2010–18 PM to the total deaths in 2010–18 ascertained from linkage of mortality data sources [16]. Unaltered: Unfilled markers. PM and rates are based on unaltered certification PM trend assessed by Mantel–Haenszel χ2 test for trend; rates trend assessed by Poisson regression †Trend in alternative PM or rate over triennia between 2001 and 2018 (*p* < 0.05, df = 5) *Trend in alternative PM or rate over triennia within 2001–09 or 2010–18 (*p* < 0.05, df = 2) ‡Trend in unaltered PM or rate over triennia between 2001 and 2018 (*p* < 0.05, df = 5) ^Trend in unaltered PM or rate over triennia within 2001–09 or 2010–18 (*p* < 0.05, df = 2)

Alternative age-standardised CVD mortality rates in men aged 35–59 years in 2001–09 and 2010–18 were 7–15% higher than the respective unaltered period rates, though remained around 3/10^3^ (95%CIs: 2.9–3.7/10^3^). In women aged 35–59 years, alternative CVD mortality rates in both periods were 16% and 12% higher than the unaltered period CVD rates at 1.5/10^3^ (95%CIs: 1.3–1.8/10^3^) and 1.1/10^3^ (95%CIs: 0.9–1.3/10^3^), respectively. In older adults, 60–74 years, the alternative period CVD mortality rates were 12–28% higher than the unaltered CVD rates. In both sexes, triennial CVD mortality rates demonstrated no significant trend over 2001–18 in ages 35–59 years (Fig. 1, panels A and C), whereas CVD rates declined in the older ages (Fig. 2, panels A and C).

### Diabetes mellitus mortality

Based on unaltered certification, of all deaths in men aged 35–59 years, diabetes accounted for 15% (95%CI: 13–17%) in 2001–09 and 19% (95%CI: 17–21%) in 2010–18; diabetes proportional mortality in 60–74 years was similar across both periods (15–22%). Alternative diabetes proportional mortality in men was lower than the unaltered proportional mortality across both periods by 3–6% points in aged 35–59 years and in 60–74 years by 5–8% points (Table 1). In women across both periods, the unaltered diabetes proportional mortality was 20–24% in ages 35–59 years (95%CIs: 17–23% and 21–27%); in ages 60–74 years, unaltered diabetes proportional mortality was 32% in both periods (95%CIs: 29–36% and 30–35%). Alternative diabetes proportional mortality in women was lower than the unaltered proportional mortality by 4–5% points in aged 35–59 years and in 60–74 years by 7% (Table 2). Both unaltered and alternative diabetes proportional mortality demonstrated increasing trends in both sexes and age groups (Figs. 1 and 2, panels B and D).

Alternative age-standardised diabetes mortality rates were significantly lower than the unaltered rates across all groups. In ages 35–59 years, the alternative diabetes rates in 2001–09 and 2010–18 were 20–34% lower in men (*p* < 0.0001) at 1.1/10^3^ (95%CI: 0.9–1.3/10^3^) and 1.3/10^3^ (95%CI: 1.1–1.5/10^3^) respectively; while in women were 23% and 15% lower (*p* = 0.013), at 1.1/10^3^ (95%CI: 0.9–1.3/10^3^) and 1.4/10^3^ respectively (95%CI: 1.2–1.7/10^3^). In older adults, differences between alternative and unaltered rates were larger.

Over 2001–18, triennial diabetes mortality rates demonstrated significant increasing trends in men aged 35–59 years (unaltered, *p* < 0.0001; alternative, *p* = 0.0007) (Fig. 1, panels A and C) and 60–74 years (unaltered, *p* < 0.0001; alternative, *p* = 0.0004) (Fig. 2, panels A and C); and in women, increased overall between 2001–03 and 2016–18 (unaltered *p* = 0.0015; alternative *p* = 0.014).

## Discussion

When diabetes and hypertension, without potentially fatal disease-specific complications, were reassigned as contributing causes of death on the MCCD, adult diabetes mortality levels over 2001–18 were lower than the diabetes mortality levels based on unaltered certification, while CVD mortality levels, conversely, were substantially higher than the unaltered certified CVD mortality levels. In adults aged 35–59 years, diabetes mortality based on unaltered certification increased while CVD mortality demonstrated no substantial trend. In older adults, diabetes mortality increased while CVD mortality decreased in both certification scenarios.

The findings reported here have important public health implications for refining prevention and control approaches for these two major causes of premature adult mortality in Tonga. The modifiable risk factors for atherosclerotic CVD (including stroke) are more numerous and diverse than those for adult-onset diabetes mellitus (type 2 diabetes), and include hypertension, tobacco smoking, and dyslipidaemia [27]. There is strong evidence for the efficacy and effectiveness of interventions targeting these risk factors in both individuals [28–32] and in populations [33–36] for disease prevention and control. CVD prevention and control focuses strongly on diet including reduced saturated and trans fats and reduced salt, and tobacco use, because reversal of diabetes and sustained optimal glycaemic control is difficult to achieve and evidence of effectiveness is mixed [37, 38]. CVD is a major cause of death in people with diabetes. A serious potential consequence of artefactual inflation of diabetes mortality indices is a disproportionate focus on the prevention and control of diabetes specifically, rather than on CVD risks more broadly. Further, in settings of limited health system resources, the apparent (and unexplained) decline in CVD mortality may provide impetus to reduce expenditures and efforts to prevent CVD.

The alternative assignment of UCoD in the present study suggests that a considerable proportion of the increases in deaths attributed to diabetes, and corresponding decreases in deaths attributed to CVD, has resulted from assignment of UCoD from CVD to diabetes without recorded potentially fatal complications. There is evidence in other settings that this is a consequence of changes in certification and coding conventions over time when diabetes is reported on the MCCD [13–15].

Regarding certification practices, likely contributors to certifier reporting of diabetes and the increasing secular trend in diabetes mortality (based on both unaltered and alternative cause-of-death certification) in Tonga include: increased testing and diagnosis of diabetes; awareness of diabetes from government and development partner activities [7–9, 39–41]; and, reported increases in diabetes burden in the community. Secular increases in type 2 diabetes in Tonga (1.9% increase every five years) have been documented from population surveys [5, 7–9] in adults aged 25–64 years [42], in correlation with increases in obesity (2.7% per five years) [5].

The 2017 Tonga STEPwise approach to NCD surveillance (STEPS) survey among adults aged 18–69 years indicated that 77% were obese (BMI ≥ 30 kg/m^2^) [9]. The prevalence of smoking/tobacco use, hypertension, and blood cholesterol have also been documented in Tonga’s STEPS surveys (2004, 2012, 2017) [7–9], however adjustment for sampling and measurement biases is needed to permit valid comparisons over time, as other analyses demonstrate [5, 43, 44]. The increased risk and occurrence of CVD and related risk factors among people with diabetes [7–9, 39] can make cause-of-death certification of diabetes problematic [45, 46]. Certifiers may have different understandings of the causal relationships between diabetes and CVD, CVD risk factors, and other conditions. Lu et al. (2005) found that half of the difference in the age-standardised diabetes mortality rates between Taiwan and other countries (Australia and Sweden) was an artifact of local certifier preferences to report diabetes in Part 1 of the MCCD [15].

Regarding coding conventions, misplacement of uncomplicated and non-fatal diabetes in Part 1 of the MCCD may result in the inappropriate selection of diabetes as the UCoD due to anomalies in the ICD-10 coding rules related to diabetes and its complications. The ICD-10 coding rules accept common CVDs, including IHD, hypertensive diseases, and various other conditions as being caused by diabetes, if these conditions are reported in Part 1 of the MCCD; this includes diabetes with non-fatal complications, diabetes ‘without complications’ or diabetes ‘with unspecified complications’ [11, 12] (see Additional file 3, Table S2). These specific rules appear inconsistent with current medical understanding, except for the uncommon diabetic cardiomyopathy [47].

Possible substitution of diabetes for CVD as the UCoD over time due to certification and coding artefacts has been demonstrated elsewhere [13–15]. Adair et al*.* (2010) demonstrated an increase in diabetes reporting in Part 1 over Part 2 over 1999–2006 in Australia among MCCD with both diabetes and CVD recorded [14]. Without this increase, the reported diabetes mortality (as the UCoD) in 2006 would have been 12% lower. Morrell *et al.* (2019) identified significant increases in type 2 diabetes proportional mortality, and concurrent and approximately equal decreases in CVD proportional mortality in Fiji (over 2001–2012) and Mauritius (over 2005–2014), which coincided with the changeover from ICD-9 to ICD-10 coding in these countries [13]. The ICD-9 coding rules allowed coders to relocate diabetes without evident fatal complications from Part 1 to Part 2 of the MCCD. The ICD-10 coding rule of strict retention of diabetes in Part 1 appears to have contributed to over-assignment of diabetes as the UCoD.

Variations in reporting diabetes on the MCCD with consequential application of ICD-10 coding of diabetes hampers valid international comparisons of diabetes mortality rates and trends. Specific international standard guidelines on cause-of-death certification for diabetes-related deaths are needed [48, 49]. Training of local staff would be needed to institutionalise updated standard ICD guidelines in countries.

This study combines and uses the most reliable cause-of-death data from multiple sources in Tonga, including MCCDs, community nursing reports and hospital discharge data to strengthen the validity of the MCCD data and accuracy of the final UCoD assignment. Given the uncertain quality of the cause-of-death data across these sources, one reference standard would not be appropriate in this setting, as previously demonstrated [2].

The present study is the first to review the original certification of all MCCDs and cause-of-death data sources for Tonga over 2001–18, use automated ICD-10 coding to efficiently standardise assignment of UCoD, and analyse and assess the impact of inappropriate certification of non-fatal diabetes and hypertension in Part 1 of the MCCD.

Study limitations include possible overestimation or underestimation of diabetes mortality in the alternative cause-of-death certification. Regarding the former, unspecified chronic renal disease or chronic renal failure coincident with diabetes in Part 1 may not have been caused by diabetes. In this case, the retention of diabetes in Part 1 may overestimate ‘diabetes with renal complications’ as the UCoD. Moreover, other plausible causes of death may not have been recorded on some MCCD where diabetes was the only condition recorded. An example of possible underestimation of diabetes mortality is that the certifier may have recorded ‘diabetes’ without also recording the occurrence of potentially fatal diabetic complications. More complete recording and better characterisation of disease-specific complication(s) are needed on the MCCD. Improvements in the completeness of MCCDs in adult age groups may limit comparability of mortality rates over the study period. However, the proportion of ill-defined or unknown causes in the 2001–09 MCCDs were comparable to the 2010–18 combined cause-of-death data sources (see Tables 1 and 2).

In conclusion, the reporting of diabetes without potentially fatal complications in Part 1 of the MCCD has led to over-estimation of diabetes mortality and under-estimation of CVD mortality in Tonga. Accurate certification of diabetes as a direct cause (Part 1) or contributory cause (Part 2) is needed to ensure that valid UCoD are assigned for public health purposes. This study underscores the importance of proper and sufficient completion of an ICD-compliant MCCD, combined with consistent standardised and correct application of ICD-10 coding rules. Records containing multiple cause(s) of death can be pre-processed to correct inappropriate certification of the UCoD and improve understanding of the causes of premature mortality in Tonga.

### Supplementary Information

**Additional file 1:** **Figure S1.** Medical certificates of cause of death: World Health Organization International Form (A) and Tongan Form (B).

**Additional file 2:** **Table S1.** Appropriate medical certification of cause of death involving diabetes and hypertension: criteria based on published scientific literature.

**Additional file 3:** **Figure S2.** Examples of different causal sequences involving diabetes on medical certificates of cause of death, and selection of underlying cause of death based on ICD-10 coding rules.

**Additional file 4:** **Figure S3.** Examples of different causal sequences involving hypertension on medical certificates of cause of death, and selection of underlying cause of death based on ICD-10 coding rules.

**Additional file 5:** **Table S2.** Conditions accepted as due to diabetes (E10–E14) when diabetes code E10.9–E14.9 is reported in Part 1 according to ICD-10 coding rules^a^.

**Additional file 6:** **Table S3.** Conditions accepted as due to essential (primary) hypertension (I10) when hypertension reported in Part 1 according to ICD-10 coding rules^a^.

**Additional file 7:** Estimation of the numerators for the cause-specific mortality rates The proportional mortality by cause and estimated total number of deaths by cause in 2010–18 (or triennia 2010–12, 2013–15, 2016–18) were defined as follows.

## Data Availability

The mortality dataset analysed during the current study are not publicly available due to ethical restriction (contains personal identifiable information) and are held on secure servers with restricted access. Access was obtained through the approval of the research ethics committee and data custodians at the Ministry of Health, Tonga. Data are however available from the corresponding author on reasonable request and with permission of the Ministry of Health, Tonga.
