# Supplementary material for: Effects of inappropriate cause-of-death certification on mortality from cardiovascular disease and diabetes mellitus in Tonga
Source: BMC Public Health. 2023 Dec 1;23:2381. doi: 10.1186/s12889-023-17294-z (PMC10691179; doi:10.1186/s12889-023-17294-z)
Supplement: Supplementary file 1 — Additional file 1: Figure S1. Medical certificates of cause of death: World Health Organization International Form (A) and Tongan Form (B). [file 12889_2023_17294_MOESM1_ESM.docx]

Figure S1: Medical certificates of cause of death: World Health Organization International Form (A) and Tongan Form (B)

| **A) International form of medical certificate of cause of death (2016 version)[1]**  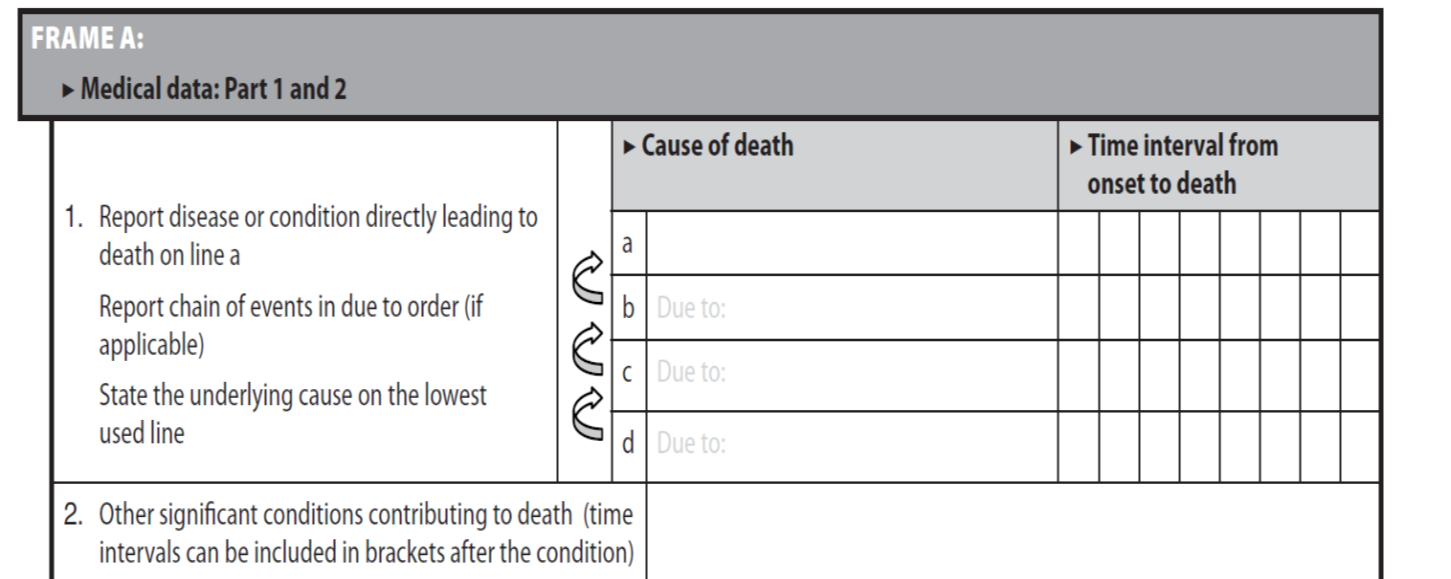 |
| --- |
| [1] Source: World Health Organization. Annex 7.1 International form of medical certificate cause of death. In: International statistical classification of diseases and related health problems, 10th revision, Volume 2 Instruction Manual. 5th ed. Geneva: World Health Organization, 2016.  **B) Tongan medical certificate of cause of death**  **~~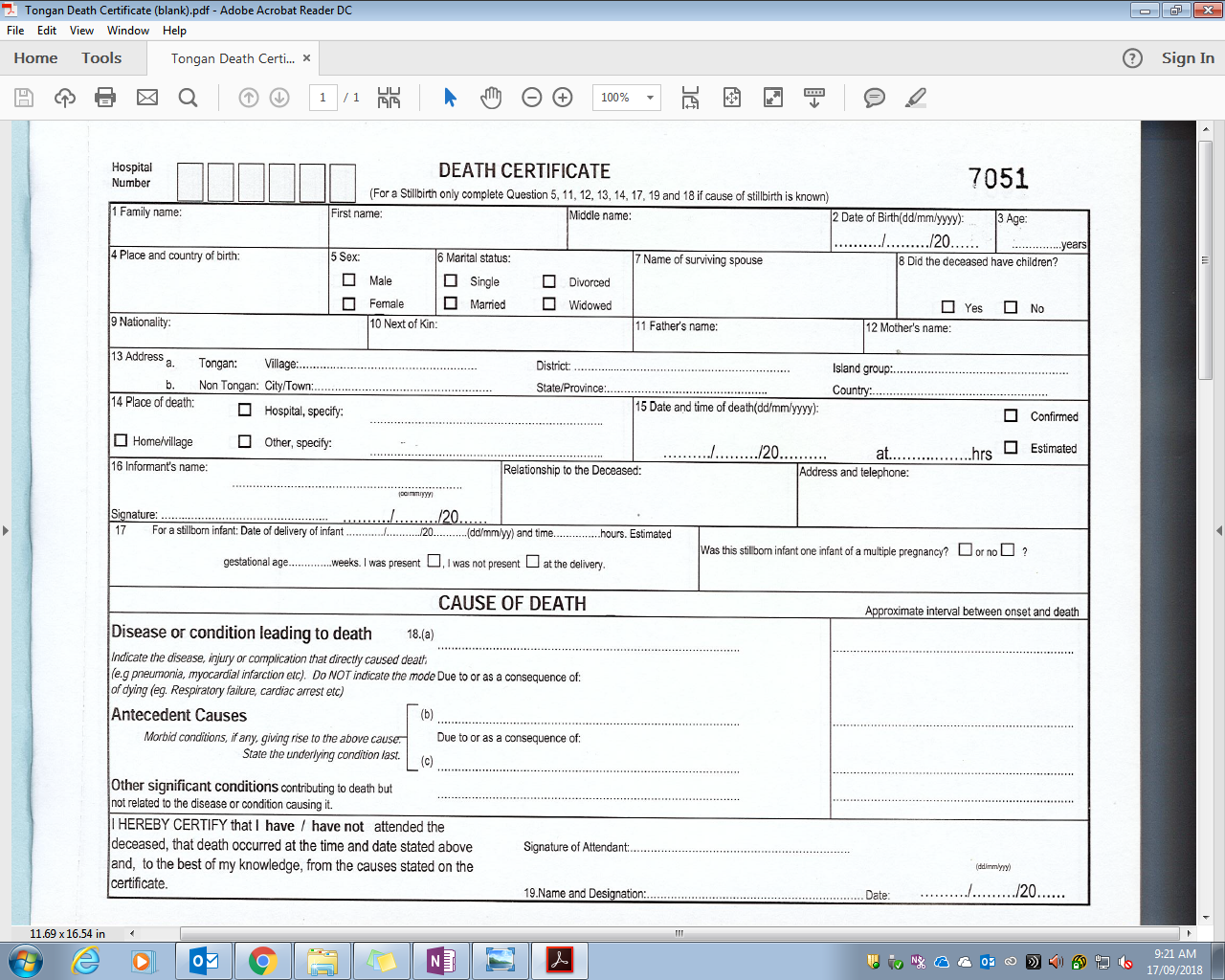~~** |
