# Supplementary material for: Effects of inappropriate cause-of-death certification on mortality from cardiovascular disease and diabetes mellitus in Tonga
Source: BMC Public Health. 2023 Dec 1;23:2381. doi: 10.1186/s12889-023-17294-z (PMC10691179; doi:10.1186/s12889-023-17294-z)
Supplement: Supplementary file 2 — Additional file 2: Table S1. Appropriate medical certification of cause of death involving diabetes and hypertension: criteria based on published scientific literature. [file 12889_2023_17294_MOESM2_ESM.docx]

Table S1. Appropriate medical certification of cause of death involving diabetes and hypertension: criteria based on published scientific literature

| **ICD-10 codes in Part 1[1]** | **Rationale and evidence** |
| --- | --- |
| **E1X.0, E1X.1**  ***or***  **E1X.X *plus:***  **E16.0, E16.1, E16.2**  Diabetes (type 1, type 2, or unspecified type) with coma, ketoacidosis; or hypoglycaemia also listed in Part 1 | ***Retain diabetes code in Part 1***  Acute metabolic complications including diabetic ketoacidosis, nonketotic hyperosmolar coma from severely high blood glucose levels, and hypoglycaemic coma from severely low blood glucose – are potentially fatal without treatment.  [2] |
| **E1X.2**  ***or***  **E1X.X *plus:***  **N18.0, N18.4, N18.5, N18.9, N19**  Diabetes (type 1, type 2, or unspecified type) with renal (kidney) complications, including chronic renal disease or renal failure, also listed in Part 1 | ***Retain diabetes code in Part 1***  Chronic renal complications of diabetes develop from hyperglycaemia-induced damage to renal vessels and cells. Diabetic renal disease, characterised by proteinuria and decline in glomerular filtration rate, can progress to end-stage renal failure. Without adequate treatment (with dialysis or transplant), the resulting uraemia is fatal.  [2-5] |
| **E1X.5**  Diabetes (type 1, type 2, or unspecified type) with peripheral circulatory complications also listed in Part 1 | ***Retain diabetes code in Part 1***  Diabetic peripheral angiopathy can develop from the direct effects of hyperglycaemia on microvasculature, causing ischaemia and potentially fatal gangrene. This may be exacerbated by immune dysfunction from the effects of hyperglycaemia on functions of immune cells (granulocytes and T-cells). Lower leg amputation is commonly required. [2, 3] |
| **E1X.6 *plus:***  **L97; L02.X, L03.X, L08.9, L89.X;**  **A41.X**  Diabetes (type 1, type 2, or unspecified type) with chronic (foot, leg) ulcer or other skin complications, with sepsis, also listed in Part 1 | ***Retain diabetes code in Part 1***  The effects of hyperglycaemia on microvasculature, neurons, and cellular immunity can lead to chronic ulceration in the lower extremities (e.g. foot, leg). Peripheral neuropathy from nerve ischaemia can manifest as sensorimotor dysfunction (e.g. paresthesia, dysesthesia, and loss of proprioception, and sensitivities to vibrations and temperature). The resultant reduced perception of trauma, even minor, and impaired wound healing can lead to ulceration. Diabetic foot ulceration can progress to limb-threatening infection and potentially fatal sepsis. Lower leg amputation is commonly required. [2, 3] |
| **E1X.X *plus:***  **I42.9 or I50.X**  ***in absence of:***  **I42.1, I42.2, I42.3, I42.4, I42.5, I42.6, I42.7**  **I00–I41, I44–I99**  Diabetes (type 1, type 2, or unspecified type) with unspecified cardiomyopathy, or (congestive) heart failure also listed in Part 1, in the absence of other known causes of cardiomyopathy including alcoholic cardiomyopathy, drugs, or external agents, or other cardiac conditions | ***Retain diabetes code in Part 1***  Diabetic cardiomyopathy refers to damage to the myocardium (heart muscle) in the absence of cardiovascular conditions including coronary artery disease, valvular disease, and risk factors, such as hypertension and dyslipidaemia. This can cause ventricular dysfunction and diastolic dysfunction, and progress to potentially fatal heart failure.  [3, 6] |
| **E1X.6, E1X.7, E1X.8**  Diabetes (type 1, type 2, or unspecified type) with other related complication(s) also listed in Part 1, either specified (e.g. musculoskeletal, skin complications, hypoglycaemia, or hyperglycaemia) or unspecified | Code E1X.6 represents ‘diabetes with other specified complications’, E1X.7 represents ‘diabetes with multiple complications’, and E1X.8 represents ‘diabetes with unspecified complications’. Five-character codes under these subcategories denote a range of complications, both non-fatal and potentially fatal.  E1X.61: with diabetic arthropathy; E1X.62: with skin complications (e.g. skin ulcer); E1X.63: with oral complications; E1X.64: with hypoglycaemia; E1X.65: with hyperglycaemia; E1X.69: with ‘other specified complications’.  Because Iris does not accept five-character codes, diabetes codes E1X.6, E1X.7, and E1X.8 are retained in Part 1 if they are accompanied by other diabetes codes and any retention criteria above are met. Certification with codes E1X.6, E1X.7, and E1X.8 were reviewed.  [2, 3] |
| **E10.6, E10.7**  Type 1 diabetes with related complication(s) specified | ***Retain diabetes code in Part 1***  [7] |
| **I10 *plus:***  **I51.4–I51.9, I50.X**  Essential (primary) hypertension with heart diseases, heart failure including congestive heart failure also listed in Part 1 | ***Retain hypertension code in Part 1***  Chronic hypertension can lead to alterations to the architecture and composition of the myocardium that result in left ventricular hypertrophy. Complications include myocarditis, myocardial degeneration, cardiomegaly, and acute or chronic carditis, all of which can progress to potentially fatal (congestive) heart failure.  [8, 9] |
| **I10 *plus:***  **N18.0, N18.4, N18.5, N18.9, N19**  Essential (primary) hypertension with chronic renal disease or chronic renal failure also listed in Part 1 | ***Retain hypertension code in Part 1***  Chronic and severe hypertension can cause vascular and glomerular injury, and ischaemic glomeruli. Without adequate therapy, renal disease can progress rapidly to renal failure.  [10] |
| **E1X.3, E1X.4, E1X.9, I10**  No criteria above are met | ***Reallocate diabetes and/or hypertension code in Part 2***  Diabetic ophthalmic complications (cataract, retinopathy) (E1X.3) and nerve complications (neuropathy) (E1X.4) are generally non‑fatal, although quality of life can be significantly reduced.  Diabetic retinopathy due to the effects of hyperlipaemia on vascular integrity can progress to blindness without treatment.  Diabetic peripheral sensorimotor neuropathy can exacerbate distal vascular insufficiency and lead to ulceration, gangrene, infection, and sepsis, as previously described above for E1X.5 and E1X.6. Diabetic neuropathy may also present as pain, hyper-sensation, muscle weakness and atrophy, motor dysfunction, and autonomic neuropathy (e.g. gastroparesis).  [2, 3]  Code E1X.9 signifies well-controlled diabetes without hypoglycaemia, hyperglycaemia, or other diabetes-specific complications.  Code I10 signifies essential hypertension, and alone, denotes no attendant hypertensive complications.  Ischaemic heart disease and myocardial infarction, and cerebrovascular disease and stroke, are considered major ‘macrovascular’ complications of diabetes. However, the underlying pathology in these cases is generally accepted as atherosclerosis. Atherosclerosis arises from a combination of multiple cellular and molecular processes including dyslipidaemia and hypertension that commonly accompany diabetes, not from chronic hyperglycaemia alone. Hyperglycaemia is considered to accelerate atherosclerosis but is not its underlying cause.  [11, 12] |

‘X’ within ICD-10 codes denotes any possible numeric character according to the International Statistical Classification of Diseases and Related Health Problems, 10th revision (ICD-10) [13]

## **References**

1. World Health Organization. International statistical classification of diseases and related health problems, 10th revision, Volume 2 Instruction Manual. 5th ed. Geneva: World Health Organization, 2016.

2. Brutsaert EF. Diabetes mellitus. The Merck MSD Manual Professional Version. New Jersey: Merck & Co. Inc., 2020. https://www.msdmanuals.com/professional/endocrine-and-metabolic-disorders/diabetes-mellitus-and-disorders-of-carbohydrate-metabolism/diabetes-mellitus-dm. Accessed 18 October 2021

3. Forbes JM, Cooper ME. Mechanisms of diabetic complications. Physiol Rev 2013;93(1):137-88.

4. Gallagher H, Suckling R. Diabetic nephropathy: where are we on the journey from pathophysiology to treatment? Diabetes Obes Metab 2016;18(7):641-47.

5. Mogensen CE, Christensen CK, Vittinghus E. The stages in diabetic renal disease. With emphasis on the stage of incipient diabetic nephropathy. Diabetes 1983;32 Suppl 2:64-78.

6. Boudina S, Abel ED. Diabetic Cardiomyopathy Revisited. Circulation 2007;115(25):3213-23.

7. Atkinson MA, Eisenbarth GS, Michels AW. Type 1 diabetes. Lancet 2014;383(9911):69-82.

8. Bakris GL. Hypertension. The Merck MSD Manual Professional Version. New Jersey: Merck & Co. Inc., 2021. https://www.msdmanuals.com/professional/cardiovascular-disorders/hypertension/hypertension. Accessed 18 October 2021

9. Tackling G, Borhade MB. Hypertensive Heart Disease. Treasure Island: StatPearls Publishing. 2022. https://www.ncbi.nlm.nih.gov/books/NBK539800/. Accessed 27 June 2022

10. Bidani AK, Griffin KA. Pathophysiology of Hypertensive Renal Damage. Hypertension 2004;44(5):595-601.

11. Aronson D, Rayfield EJ. How hyperglycemia promotes atherosclerosis: molecular mechanisms. Cardiovasc Diabetol 2002;1(1):1.

12. Fuller J, Stevens L, Wang S. Risk factors for cardiovascular mortality and morbidity: The WHO multinational study of vascular disease in diabetes. Diabetologia 2001;44(2):S54-S64.

13. World Health Organization. International statistical classification of diseases and related health problems, 10th revision, Volume 1 Tabular List. 5th ed. Geneva: World Health Organization, 2016
