# Supplementary material for: Effects of inappropriate cause-of-death certification on mortality from cardiovascular disease and diabetes mellitus in Tonga
Source: BMC Public Health. 2023 Dec 1;23:2381. doi: 10.1186/s12889-023-17294-z (PMC10691179; doi:10.1186/s12889-023-17294-z)
Supplement: Supplementary file 3 — Additional file 3: Figure S2. Examples of different causal sequences involving diabetes on medical certificates of cause of death, and selection of underlying cause of death based on ICD-10 coding rules. [file 12889_2023_17294_MOESM3_ESM.docx]

Figure S2: Examples of different causal sequences involving **diabetes** on medical certificates of cause of death, and selection of underlying cause of death based on ICD-10 coding

| **Diabetes retained in Part 1** | **Diabetes reported in Part 1 – inappropriate** | **Diabetes reallocated in Part 2** |
| --- | --- | --- |
| \| **Part 1 and 2** \| \| \| **ICD-10 codes** \| \| --- \| --- \| --- \| --- \| \| 1 \| **Cause of death** \| \| \| a  b  c  d \| Diabetic hyperosmolar coma Type 2 diabetes mellitus \| E110 E119 \| \| 2 \| \|  \|  \| \| **UCoD** \| \| **Type 2 diabetes mellitus with coma** \| **E110** \| | \| **Part 1 and 2** \| \| \| **ICD-10 codes** \| \| --- \| --- \| --- \| --- \| \| 1 \| **Cause of death** \| \| \| a  b  c  d \| Myocardial infarction Complication of uncontrolled type 2 diabetes \| I219  E118 \| \| 2 \| \| Hypertension \| I10 \| \| **UCoD** \| \| **Type 2 diabetes mellitus with multiple complications** \| **E117** \| | \| **Part 1 and 2** \| \| \| **ICD-10 codes** \| \| --- \| --- \| --- \| --- \| \| 1 \| **Cause of death** \| \| \| a  b  c  d \| Myocardial infarction \| I219 \| \| 2 \| \| Complication of uncontrolled type 2 diabetes \| E118 \| \| **UCoD** \| \| **Acute myocardial infarction, unspecified** \| **I219** \| |
| \| **Part 1 and 2** \| \| \| **ICD-10 codes** \| \| --- \| --- \| --- \| --- \| \| 1 \| **Cause of death** \| \| \| a  b  c  d \| Diabetic ketoacidosis  Diabetes \| E141 E149 \| \| 2 \| \|  \|  \| \| **UCoD** \| \| **Unspecified diabetes mellitus with ketoacidosis** \| **E141** \| | \| **Part 1 and 2** \| \| \| **ICD-10 codes** \| \| --- \| --- \| --- \| --- \| \| 1 \| **Cause of death** \| \| \| a  b  c  d \| Left Ischemic Stroke  Diabetic retinopathy  Type 2 Diabetes \| I639 E113 E119 \| \| 2 \| \|  \|  \| \| **UCoD** \| \| **Type 2 diabetes mellitus with multiple complications** \| **E117** \| | \| **Part 1 and 2** \| \| \| **ICD-10 codes** \| \| --- \| --- \| --- \| --- \| \| 1 \| **Cause of death** \| \| \| a  b  c  d \| Left Ischemic Stroke \| I639 \| \| 2 \| \| Diabetic retinopathy, Type 2 Diabetes \| E113, E119 \| \| **UCoD** \| \| **Left Ischemic Stroke** \| **I639** \| |
| \| **Part 1 and 2** \| \| \| **ICD-10 codes** \| \| --- \| --- \| --- \| --- \| \| 1 \| **Cause of death** \| \| \| a  b  c  d \| Renal failure  Diabetic nephropathy  Type 2 diabetes mellitus \| N19 E112 E119 \| \| 2 \| \| Hypertension \| I10 \| \| **UCoD** \| \| **Type 2 diabetes mellitus with renal complications** \| **E112** \| | \| **Part 1 and 2** \| \| \| **ICD-10 codes** \| \| --- \| --- \| --- \| --- \| \| 1 \| **Cause of death** \| \| \| a  b  c  d \| Haemorrhagic stroke  Type 2 diabetes mellitus \| I619  E119 \| \| 2 \| \|  \|  \| \| **UCoD** \| \| **Type 2 diabetes mellitus with other specified complication** \| **E116** \| | \| **Part 1 and 2** \| \| \| **ICD-10 codes** \| \| --- \| --- \| --- \| --- \| \| 1 \| **Cause of death** \| \| \| a  b  c  d \| Haemorrhagic stroke \| I619 \| \| 2 \| \| Type 2 diabetes mellitus \| E119 \| \| **UCoD** \| \| **Haemorrhagic stroke** \| **I619** \| |
| \| **Part 1 and 2** \| \| \| **ICD-10 codes** \| \| --- \| --- \| --- \| --- \| \| 1 \| **Cause of death** \| \| \| a  b  c  d \| Severe sepsis  Diabetic gangrene Uncontrolled type 2 diabetes \| A419 E115 E116 \| \| 2 \| \|  \|  \| \| **UCoD** \| \| **Type 2 diabetes mellitus with multiple complications** \| **E117** \| | \| **Part 1 and 2** \| \| \| **ICD-10 codes** \| \| --- \| --- \| --- \| --- \| \| 1 \| **Cause of death** \| \| \| a  b  c  d \| Cardiac arrest  Pulmonary embolism, acute cor pulmonale  Complication of type 2 diabetes \| I469 I260  E118 \| \| 2 \| \|  \|  \| \| **UCoD** \| \| **Type 2 diabetes mellitus with unspecified complications** \| **E118** \| | \| **Part 1 and 2** \| \| \| **ICD-10 codes** \| \| --- \| --- \| --- \| --- \| \| 1 \| **Cause of death** \| \| \| a  b  c  d \| Cardiac arrest  Pulmonary embolism, acute cor pulmonale \| I469 I260 \| \| 2 \| \| Complication of type 2 diabetes \| E118 \| \| **UCoD** \| \| **Pulmonary embolism, acute cor pulmonale** \| **I260** \| |
| \| **Part 1 and 2** \| \| \| **ICD-10 codes** \| \| --- \| --- \| --- \| --- \| \| 1 \| **Cause of death** \| \| \| a  b  c  d \| Foot ulcer  Diabetic arthropathy  Type 1 diabetes \| L97 E106 E109 \| \| 2 \| \|  \|  \| \| **UCoD** \| \| **Type 1 diabetes with multiple complications** \| **E107** \| | \| **Part 1 and 2** \| \| \| **ICD-10 codes** \| \| --- \| --- \| --- \| --- \| \| 1 \| **Cause of death** \| \| \| a  b  c  d \| Acute myocardial infarction Chronic ischaemic heart disease Diabetic neuropathy \| I219 I259 E114 \| \| 2 \| \|  \|  \| \| **UCoD** \| \| **Type 2 diabetes mellitus with multiple complications** \| **E117** \| | \| **Part 1 and 2** \| \| \| **ICD-10 codes** \| \| --- \| --- \| --- \| --- \| \| 1 \| **Cause of death** \| \| \| a  b  c  d \| Acute myocardial infarction Chronic ischaemic heart disease \| I219 I259 \| \| 2 \| \| Diabetic neuropathy \| E114 \| \| **UCoD** \| \| **Acute myocardial infarction** \| **I219** \| |
| \| **Part 1 and 2** \| \| \| **ICD-10 codes** \| \| --- \| --- \| --- \| --- \| \| 1 \| **Cause of death** \| \| \| a  b  c  d \| End-stage renal failure Nephritic syndrome  Type 2 diabetes mellitus \| N189 N05 E119 \| \| 2 \| \|  \|  \| \| **UCoD** \| \| **Type 2 diabetes with renal complications** \| **E112** \| | \| **Part 1 and 2** \| \| \| **ICD-10 codes** \| \| --- \| --- \| --- \| --- \| \| 1 \| **Cause of death** \| \| \| a  b  c  d \| Left ventricular failure  Diabetic retinopathy Hypertension \| I501 E143  I10 \| \| 2 \| \|  \|  \| \| **UCoD** \| \| **Unspecified diabetes mellitus with multiple complications** \| **E147** \| | \| **Part 1 and 2** \| \| \| **ICD-10 codes** \| \| --- \| --- \| --- \| --- \| \| 1 \| **Cause of death** \| \| \| a  b  c  d \| Left ventricular failure Hypertension \| I501 I10 \| \| 2 \| \| Diabetic retinopathy \| E143 \| \| **UCoD** \| \| **Hypertensive heart disease with heart failure** \| **I110** \| |
| \| **Part 1 and 2** \| \| \| **ICD-10 codes** \| \| --- \| --- \| --- \| --- \| \| 1 \| **Cause of death** \| \| \| a  b  c  d \| Sepsis  Chronic skin ulcer  Diabetes \| A419  L97 E149 \| \| 2 \| \|  \|  \| \| **UCoD** \| \| **Diabetes with multiple complications** \| **E147** \| | \| **Part 1 and 2** \| \| \| **ICD-10 codes** \| \| --- \| --- \| --- \| --- \| \| 1 \| **Cause of death** \| \| \| a  b  c  d \| Respiratory failure  Lung cancer  Uncontrolled type 2 diabetes \| J969 C349 E116 \| \| 2 \| \|  \|  \| \| **UCoD** \| \| **Malignant neoplasm of lung** \| **C349*** \| | \| **Part 1 and 2** \| \| \| **ICD-10 codes** \| \| --- \| --- \| --- \| --- \| \| 1 \| **Cause of death** \| \| \| a  b  c  d \| Respiratory failure  Lung cancer \| J969 C349 \| \| 2 \| \| Uncontrolled type 2 diabetes \| E116 \| \| **UCoD** \| \| **Malignant neoplasm of lung** \| **C349*** \| |
| \| **Part 1 and 2** \| \| \| **ICD-10 codes** \| \| --- \| --- \| --- \| --- \| \| 1 \| **Cause of death** \| \| \| a  b  c  d \| Hypoglycemia  Type 1 diabetes \| E162 E109 \| \| 2 \| \|  \|  \| \| **UCoD** \| \| **Type 1 diabetes mellitus with other specified complications** \| **E106** \| | \| **Part 1 and 2** \| \| \| **ICD-10 codes** \| \| --- \| --- \| --- \| --- \| \| 1 \| **Cause of death** \| \| \| a  b  c  d \| Fracture of femur  Accidental fall  Uncontrolled type 2 diabetes \| S723 W19 E116 \| \| 2 \| \|  \|  \| \| **UCoD** \| \| **Unspecified fall** \| **W19*** \| | \| **Part 1 and 2** \| \| \| **ICD-10 codes** \| \| --- \| --- \| --- \| --- \| \| 1 \| **Cause of death** \| \| \| a  b  c  d \| Fracture of femur Accidental fall \| S723  W19 \| \| 2 \| \| Uncontrolled type 2 diabetes \| E116 \| \| **UCoD** \| \| **Unspecified fall** \| **W19*** \| |
| \| **Part 1 and 2** \| \| \| **ICD-10 codes** \| \| --- \| --- \| --- \| --- \| \| 1 \| **Cause of death** \| \| \| a  b  c  d \| Multiple chronic leg ulcers Type 2 diabetes \| L97 E119 \| \| 2 \| \|  \|  \| \| **UCoD** \| \| **Type 2 diabetes mellitus with peripheral circulatory complications** \| **E115** \| |  |  |
| \| **Part 1 and 2** \| \| \| **ICD-10 codes** \| \| --- \| --- \| --- \| --- \| \| 1 \| **Cause of death** \| \| \| a  b  c  d \| Congestive heart failure Cardiomyopathy  Type 2 diabetes \| I500 I429 E119 \| \| 2 \| \|  \|  \| \| **UCoD** \| \| **Type 2 diabetes mellitus with other specified complications** \| **E116** \| |  |  |

Abbreviation: UCoD: underlying cause of death

* When external causes or malignant neoplasms (cancers) are reported in Part 1, they are accepted as the UCoD (i.e. are not to be accepted as due to any other cause coded in other chapters), with a few exceptions, under ‘Special instructions on accepted and rejected sequences (SP3 and SP4)’ of the ICD-10 coding rules [1]

## **Reference**

1. World Health Organization. International statistical classification of diseases and related health problems, 10th revision, Volume 2 Instruction Manual. 5th ed. Geneva: World Health Organization, 2016.
