# Supplementary material for: Effects of inappropriate cause-of-death certification on mortality from cardiovascular disease and diabetes mellitus in Tonga
Source: BMC Public Health. 2023 Dec 1;23:2381. doi: 10.1186/s12889-023-17294-z (PMC10691179; doi:10.1186/s12889-023-17294-z)
Supplement: Supplementary file 4 — Additional file 4: Figure S3. Examples of different causal sequences involving hypertension on medical certificates of cause of death, and selection of underlying cause of death based on ICD-10 coding rules. [file 12889_2023_17294_MOESM4_ESM.docx]

Figure S3: Examples of different causal sequences involving **hypertension** on medical certificates of cause of death, and selection of underlying cause of death based on ICD-10 coding rules

| **Hypertension retained in Part 1** | **Hypertension reported in Part 1 – inappropriate** | **Hypertension reallocated in Part 2** |
| --- | --- | --- |
| \| **Part 1 and 2** \| \| \| **ICD-10 codes** \| \| --- \| --- \| --- \| --- \| \| **1** \| **Cause of death** \| \| \| a  b  c  d \| Myocarditis  Hypertension \| I514 I10 \| \| **2** \| \|  \|  \| \| **UCoD** \| \| **Hypertensive heart disease without heart failure** \| **I119** \| | \| **Part 1 and 2** \| \| \| **ICD-10 codes** \| \| --- \| --- \| --- \| --- \| \| 1 \| **Cause of death** \| \| \| a  b  c  d \| Sepsis  Chronic pyelonephritis Hypertension \| A419 N119 I10 \| \| 2 \| \|  \|  \| \| **UCoD** \| \| **Essential (primary) hypertension** \| **I10** \| | \| **Part 1 and 2** \| \| \| **ICD-10 codes** \| \| --- \| --- \| --- \| --- \| \| 1 \| **Cause of death** \| \| \| a  b  c  d \| Sepsis  Chronic pyelonephritis \| A419 N119 \| \| 2 \| \| Hypertension \| I10 \| \| **UCoD** \| \| **Chronic pyelonephritis** \| **N119** \| |
| \| **Part 1 and 2** \| \| \| **ICD-10 codes** \| \| --- \| --- \| --- \| --- \| \| **1** \| **Cause of death** \| \| \| a  b  c  d \| Congestive heart failure Hypertension \| I500  I10 \| \| **2** \| \|  \|  \| \| **UCoD** \| \| **Hypertensive heart disease with congestive heart failure** \| **I110** \| | \| **Part 1 and 2** \| \| \| **ICD-10 codes** \| \| --- \| --- \| --- \| --- \| \| 1 \| **Cause of death** \| \| \| a  b  c  d \| Gastrointestinal haemorrhage Peptic ulcer  Hypertension \| K922 K274 I10 \| \| 2 \| \|  \|  \| \| **UCoD** \| \| **Essential (primary) Hypertension** \| **I10** \| | \| **Part 1 and 2** \| \| \| **ICD-10 codes** \| \| --- \| --- \| --- \| --- \| \| 1 \| **Cause of death** \| \| \| a  b  c  d \| Gastrointestinal haemorrhage  Peptic ulcer \| K922  K274 \| \| 2 \| \| Hypertension \| I10 \| \| **UCoD** \| \| **Peptic ulcer with haemorrhage** \| **K274** \| |
| \| **Part 1 and 2** \| \| \| **ICD-10 codes** \| \| --- \| --- \| --- \| --- \| \| **1** \| **Cause of death** \| \| \| a  b  c  d \| Chronic kidney disease, stage 5 Hypertension \| N185  I10 \| \| **2** \| \|  \|  \| \| **UCoD** \| \| **Hypertensive renal disease with renal failure** \| **I120** \| | \| **Part 1 and 2** \| \| \| **ICD-10 codes** \| \| --- \| --- \| --- \| --- \| \| 1 \| **Cause of death** \| \| \| a  b  c  d \| Acute myocardial infarction Chronic ischaemic heart disease Hypertension \| I219 I259  I10 \| \| 2 \| \|  \|  \| \| **UCoD** \| \| **Acute myocardial infarction** \| **I219*** \| | \| **Part 1 and 2** \| \| \| **ICD-10 codes** \| \| --- \| --- \| --- \| --- \| \| 1 \| **Cause of death** \| \| \| a  b  c  d \| Acute myocardial infarction Chronic ischaemic heart disease \| I219  I259 \| \| 2 \| \| Hypertension \| I10 \| \| **UCoD** \| \| **Acute myocardial infarction** \| **I219*** \| |
| \| **Part 1 and 2** \| \| \| **ICD-10 codes** \| \| --- \| --- \| --- \| --- \| \| **1** \| **Cause of death** \| \| \| a  b  c  d \| Heart failure  Chronic kidney disease, stage 4 Hypertension \| I509 N184 I10 \| \| **2** \| \|  \|  \| \| **UCoD** \| \| **Hypertensive heart and renal disease with both heart and renal failure** \| **I132** \| | \| **Part 1 and 2** \| \| \| **ICD-10 codes** \| \| --- \| --- \| --- \| --- \| \| 1 \| **Cause of death** \| \| \| a  b  c  d \| Pedestrian fractured skull in collision with car  Myocarditis Hypertension \| V031, S029 I514  I10 \| \| 2 \| \|  \|  \| \| **UCoD** \| \| **Pedestrian injured in collision with car** \| **V031**** \| | \| **Part 1 and 2** \| \| \| **ICD-10 codes** \| \| --- \| --- \| --- \| --- \| \| 1 \| **Cause of death** \| \| \| a  b  c  d \| Pedestrian fractured skull in collision with car Myocarditis \| V031, S029 I514 \| \| 2 \| \| Hypertension \| I10 \| \| **UCoD** \| \| **Pedestrian injured in collision with car** \| **V031**** \| |
| \| **Part 1 and 2** \| \| \| **ICD-10 codes** \| \| --- \| --- \| --- \| --- \| \| **1** \| **Cause of death** \| \| \| a  b  c  d \| Hypertensive heart failure Hypertension \| I110 I10 \| \| **2** \| \|  \|  \| \| **UCoD** \| \| **Hypertensive heart disease with (congestive) heart failure** \| **I110** \| | \| **Part 1 and 2** \| \| \| **ICD-10 codes** \| \| --- \| --- \| --- \| --- \| \| 1 \| **Cause of death** \| \| \| a  b  c  d \| Heart failure  Lung carcinoma with secondary liver metastasis Hypertension \| I509 C349, C787 I10 \| \| 2 \| \|  \|  \| \| **UCoD** \| \| **Malignant neoplasm of lung** \| **C349**** \| | \| **Part 1 and 2** \| \| \| **ICD-10 codes** \| \| --- \| --- \| --- \| --- \| \| 1 \| **Cause of death** \| \| \| a  b  c  d \| Heart failure  Lung carcinoma with secondary liver metastasis \| I509 C349, C787 \| \| 2 \| \| Hypertension \| I10 \| \| **UCoD** \| \| **Malignant neoplasm of lung** \| **C349**** \| |
| \| **Part 1 and 2** \| \| \| **ICD-10 codes** \| \| --- \| --- \| --- \| --- \| \| **1** \| **Cause of death** \| \| \| a  b  c  d \| Renal failure  Hypertensive renal disease Hypertension \| N19 I129 I10 \| \| **2** \| \|  \|  \| \| **UCoD** \| \| **Hypertensive renal disease with renal failure** \| **I120** \| |  |  |

Abbreviation: UCoD: underlying cause of death

* When hypertension (I10) is mentioned with ischaemic heart disease (IHD) (codes I20–I25), IHD is to be selected as the UCoD, under ‘Special instructions on linkages and other provisions (Step M1)’ of the ICD-10 coding rules [1]

** When external causes or malignant neoplasms (cancers) are reported in Part 1, they are accepted as the UCoD (i.e. are not to be accepted as due to any other cause coded in other chapters), with a few exceptions, under ‘Special instructions on accepted and rejected sequences (SP3 and SP4)’ of the ICD-10 coding rules [1]

## **Reference**

1. World Health Organization. International statistical classification of diseases and related health problems, 10th revision, Volume 2 Instruction Manual. 5th ed. Geneva: World Health Organization, 2016.
