# Supplementary material for: Effects of inappropriate cause-of-death certification on mortality from cardiovascular disease and diabetes mellitus in Tonga
Source: BMC Public Health. 2023 Dec 1;23:2381. doi: 10.1186/s12889-023-17294-z (PMC10691179; doi:10.1186/s12889-023-17294-z)
Supplement: Supplementary file 5 — Additional file 5: Table S2. Conditions accepted as due to diabetes (E10–E14) when diabetes code E10.9–E14.9 is reported in Part 1 according to ICD-10 coding rulesa. [file 12889_2023_17294_MOESM5_ESM.docx]

Table S2: Conditions accepted as due to diabetes (E10–E14) when diabetes code E10.9–E14.9 is reported in Part 1 according to ICD-10 coding rules^a^

| **Diabetes** **without complications (E10.9–E14.9) is assigned as the underlying cause of:** | |
| --- | --- |
| Acute pericarditis (I30)^b^  Acute myocarditis (I40)^b^  Atrioventricular and left bundle-branch block (I44)^b^  Other conduction disorders (I45)^b^ | Cardiac arrest (I46)^b^  Paroxysmal tachycardia (I47)^b^  Other ill-defined heart diseases (I51.8)^b^ |
| **Diabetes with other specified complications (E10.6–E14.6) is assigned as the underlying cause of:** | |
| ***Circulatory diseases:***  Hypertensive diseases: Essential (primary) hypertension (I10); Hypertensive heart disease (I11.-)  Ischaemic heart diseases: Acute and subsequent myocardial infarction (I21 and I22)^b^; Other acute ischaemic heart disease (I24)^b^  Pulmonary embolism (I26)^b^  Other heart diseases: Endocarditis (I33.0, I38); Cardiomyopathy (I42.0, I42.9); Atrial fibrillation and flutter (I48)^b^; Other cardiac arrhythmias (I49)^b^; Heart failure (I50.-)^b^; Unspecified (I51.6)  Cerebrovascular diseases (I60–I66, I67.6–I67.8, I69)^b^  Arterial diseases: Atherosclerosis (I70.0, I70.1, I70.8, I70.9); Embolism, thrombosis (I74.2–I74.4)  Diseases of veins and lymphatics: Phlebitis/thrombophlebitis (I80.-); Embolism, thrombosis (I82.9) | ***Other specified conditions:***  Gastroenteritis (A09.-)  Sepsis (A40.- , A41.-); Bacterial infection, unspecified site (A49.-)  Mycoses (B35.-, B36.-, B37.-)  Disseminated intravascular coagulation (defibrination syndrome) (D65)  Hypoglycaemia (E16.2)  Metabolic disorders: Lipidaemia (E78.0–E78.2, E78.5); Hyperkalaemia (E87.5); Unspecified (E88.9)  Encephalitis and myelitis (G04.8, G04.9); Nervous system disorder, not elsewhere classified (G98)  Pneumonia (J12–J18)  Gastrointestinal diseases: Ulcer (K25–K28); Haemorrhage (K92.2)  Skin and subcutaneous tissue diseases: Local infections (L03.-, L08.-); Dermatitis (L30.9); Decubitus ulcer and pressure area (L89.-); Necrobiosis lipoidica (L92.1)^c^  Musculoskeletal diseases: Chondrocalcinosis (M11.2); Arthritis (M13.9)^c^; Neuralgia and neuritis (M79.2 )^c^; Necrotising fasciitis (M72.6); Unspecified (M89.9)  Urinary tract infection (N39.0)  Diabetic arthropathy (M14.2)  Neuropathic diabetic arthropathy (M14.6) |
| **Diabetes with coma (E10.0–E14.0) is assigned as the underlying cause of:** | |
| Nondiabetic hypoglycaemic coma; for unspecified hypoglycaemic coma only (E15)  Unspecified coma (R40.2)^c^ | Diabetic hyperosmolar coma  Diabetic hypoglycaemic coma |
| **Diabetes with ketoacidosis, other metabolic complications (E10.1–E14.1) is assigned as the underlying cause of:** | |
| Acidosis (E87.2)^c^  Steatosis, other specified metabolic disorder (E88.8)^c^ | Acetonaemia, other specified abnormal findings of blood chemistry R79.8^c^ |
| **Diabetes with renal complications (E10.2–E14.2) is assigned as the underlying cause of:** | |
| Nephrotic syndrome, Nephritic syndrome (N03–N05)^c^  Chronic kidney disease (N18.-)^c^  Unspecified renal failure (N19)^c^  Unspecified contracted kidney (N26)^c^  Disorder of kidney and ureter, unspecified (N28.9)^c^ | Proteinuria, unspecified (N39.1)^c^  Diabetic nephropathy (N08.3)  Intracapillary glomerulonephrosis (N08.3)  Kimmelstiel-Wilson syndrome (N08.3) |
| **Diabetes with ophthalmic complications (E10.3–E14.3) is assigned as the underlying cause of:** | |
| Iridocyclitis, unspecified (H20.9)^c^  Cataract, unspecified (H26.9)^c^  Chorioretinal inflammation, unspecified (H30.9)^c^  Retinal vascular occlusion (H34)^c^  Other retinal disorders **(**H35.0, H35.2, H35.6, H35.9)^c^ | Paralytic strabismus, unspecified (H49.9)  Blindness and low vision (H54)  Diabetic cataract (H28.0)  Diabetic retinopathy (H36.0) |
| **Diabetes with neurological complications (E10.4–E14.4) is assigned as the underlying cause of:** | |
| Other specified diseases of stomach and duodenum, for gastroparesis only (K31.8)  Other mononeuropathies (G58.-)^c^  Polyneuropathy, unspecified (G62.9)^c^  Other disorders of peripheral nervous system (G64)^c^  Myoneural disorders of muscle (G70.9)  Other primary disorders of muscles (G71.8)^c^ | Disorder of autonomic nervous system, unspecified (G90.9)^c^  Diabetic amyotrophy (G73.0)  Diabetic autonomic neuropathy (G99.0)  Diabetic mononeuropathy (G59.0)  Diabetic polyneuropathy (G63.2) |
| **Diabetes with peripheral circulatory complications (E10.5–E14.5) is assigned as the underlying cause of:** | |
| Atherosclerosis of arteries of extremities (I70.2)^c^  Peripheral vascular disease, unspecified (I73.9)^c^  Other and unspecified disorders of circulatory system, for angiopathy only (I99)  Ulcer of lower limb (L97)^c^ | Chronic ulcer of skin, not elsewhere classified (L98.4)  Gangrene, not elsewhere classified (R02)^c^  Diabetic gangrene  Diabetic peripheral angiopathy (I79.2)  Diabetic ulcer |
| **Diabetes with multiple complications (E10.7–E14.7) is assigned as the underlying cause of:** | |
| More than one of the above conditions combined | |

a E10: type 1 diabetes mellitus; E11: type 2 diabetes mellitus; E14: diabetes, unspecified. If diabetes is reported in Part 1 with any listed condition, diabetes may be selected as the underlying cause of death (Step SP3 or SP4). If the fourth character of the diabetes code is .9 (E10.9, E11.9, or E14.9), these are coded as ‘diabetes without complications’, and the fourth character is modified as shown in each subheading (Step Modification 1). If diabetes is reported on the uppermost line in Part 1, the fourth character is not modified (Step SP5) [1]. If other diabetes codes (E10.1–.8, E11.1–.8, E14.1–.8) are reported in Part 1 with any listed condition, the assigned underlying cause is coded as ‘diabetes with multiple complications’ (E10.7, E11.7, E14.7).

b Special instruction on Steps SP3 and SP4: “Accept these acute or terminal circulatory diseases as due to malignant neoplasm, diabetes or asthma” [1].

c Selection rule Step SP6: Diabetes is an ‘obvious cause’ of this condition. Diabetes may be selected as the underlying cause of death even if it is reported in Part 2 [1].

## **Reference**

1. World Health Organization. International statistical classification of diseases and related health problems, 10th revision, Volume 2 Instruction Manual. 5th ed. Geneva: World Health Organization, 2016.
