# Supplementary material for: Effects of inappropriate cause-of-death certification on mortality from cardiovascular disease and diabetes mellitus in Tonga
Source: BMC Public Health. 2023 Dec 1;23:2381. doi: 10.1186/s12889-023-17294-z (PMC10691179; doi:10.1186/s12889-023-17294-z)
Supplement: Supplementary file 6 — Additional file 6: Table S3. Conditions accepted as due to essential (primary) hypertension (I10) when hypertension reported in Part 1 according to ICD-10 coding rulesa. [file 12889_2023_17294_MOESM6_ESM.docx]

Table S3: Conditions accepted as due to essential (primary) hypertension (I10) when hypertension reported in Part 1 according to ICD-10 coding rules^a^

| ***Hypertensive heart disease with (congestive) heart failure (I11.0) is assigned the underlying cause of:*** | |
| --- | --- |
| Heart failure, except when specified as terminal or acute, sudden, or similar expressions of short duration (less than 24 hours) (I50.-) | |
| ***Hypertensive heart disease (I11.-) is assigned the underlying cause of:*** | |
| Complications and ill-defined descriptions of heart disease, except when specified as terminal or acute, sudden, or similar expressions of short duration (less than 24 hours): | Myocarditis, unspecified (I51.4)  Myocardial degeneration (I51.5);  Cardiovascular disease, unspecified (I51.6);  Cardiomegaly (I51.7);  Other ill‑defined heart diseases (I51.8);  Heart disease, unspecified (I51.9) |
| ***Essential (primary) hypertension (I10) is assigned the underlying cause of:*** | |
| Atherosclerosis (I70.-) | |
| ***Hypertensive renal disease (I12.-) is assigned the underlying cause of:*** | |
| Chronic kidney disease (N18.-)  Unspecified kidney failure (N19)  Unspecified contracted kidney (N26) | |

a Special instructions on linkages and other provisions (Step Modification 1) [1].

## **Reference**

1. World Health Organization. International statistical classification of diseases and related health problems, 10th revision, Volume 2 Instruction Manual. 5th ed. Geneva: World Health Organization, 2016.
