# Supplementary material for: Effects of inappropriate cause-of-death certification on mortality from cardiovascular disease and diabetes mellitus in Tonga
Source: BMC Public Health. 2023 Dec 1;23:2381. doi: 10.1186/s12889-023-17294-z (PMC10691179; doi:10.1186/s12889-023-17294-z)
Supplement: Supplementary file 7 — Additional file 7: Estimation of the numerators for the cause-specific mortality rates The proportional mortality by cause and estimated total number of deaths by cause in 2010–18 (or triennia 2010–12, 2013–15, 2016–18) were defined as follows. [file 12889_2023_17294_MOESM7_ESM.docx]

## ***Estimation of the numerators for the cause-specific mortality rates***

The proportional mortality by cause and estimated total number of deaths by cause in 2010–18 (or triennia 2010–12, 2013–15, 2016–18) were defined as follows:

_n_R_xi_(G) = _n_d_xi_(G) / _n_d_x_(G)

_n_$\hat{d}$_xi_ = _n_R_xi_(G) $\times$ _n_d_x_(H)

Where:
_n_R_xi_ is the proportional mortality for cause i in the age group (x, x+n)

_n_d_xi_ is the number of deaths due to cause i in the age group (x, x+n)

_n_d_x_ is the number of deaths in the age group (x, x+n)

_n_$\hat{d}$_xi_ is the estimated total number of deaths due to cause i in the age group (x, x+n)

G is the integrated cause-of-death dataset (MCCD+community nursing reports+hospital discharge) in 2010–18

H is the reconciled total (all-cause) deaths in 2010–18

The calculations for 2010–18 were performed by sex, for each 5-year age interval between ages 5 years and 75 years. The proportional mortality of the broader age groups 35–59 years and 60–74 years were also applied to minimise the uncertainty produced by small numbers of deaths in the 5-year age groups.

The proportional mortality by cause and estimated total number of deaths by cause in 2001–09 (or triennia 2001–03, 2004–06, 2007–09) were defined as follows:

_n_R_xi_(F) = _n_d_xi_(F^0^) $/$ _n_d_x_(F^0^)

_n_$\hat{k}$_x_^0^ = _n_$\hat{k}$_x_^1^(F^1^) = _n_d_x_(F^1^) $/$ _n_d_x_(H)

_n_$\hat{d}$_x_^0^ = _n_d_xi_(F^0^) $/$ _n_$\hat{k}$_x_^0^

_n_$\hat{d}$_xi_ = _n_R_xi_(F) $\times$ _n_$\hat{d}$_x_^0^

Where:
(0) is the 2001–09 period (or triennia 2001–03, 2004–06, 2007–09)

(1) is the 2010–18 period (or triennia 2010–12, 2013–15, 2016–18)

_n_R_xi_ is the proportional mortality for cause i in the age group (x, x+n)

_n_d_xi_ is the number of deaths due to cause i in the age group (x, x+n)

_n_d_x_ is the number of deaths in the age group (x, x+n)

_n_$\hat{d}$_xi_ is the estimated total number of deaths due to cause i in the age group (x, x+n)

F is the MCCD cause-of-death dataset
H is the reconciled total (all-cause) deaths in 2010–18
$\hat{k}$ is estimated completeness reported as a fraction.

The above calculations for 2001–09 were performed by sex, for each 5-year age interval between adult ages 35 and 74 years, and for the broad age groups 35–59 years and 60–74 years.
